# Editorial: Nanomaterials-based electrochemical biosensors

**DOI:** 10.3389/fbioe.2022.1091592

**Published:** 2022-12-14

**Authors:** Shimaa Eissa, Chaker Tlili, Ben Ali Mounir, Olfa Kanoun

**Affiliations:** ^1^ Department of Chemistry, Khalifa University, Abu Dhabi, United Arab Emirates; ^2^ Chongqing Institute of Green and Intelligent Technology, Chinese Academy of Sciences, Chongqing, China; ^3^ University of Sousse, Higher Institute of Applied Sciences and Technology of Sousse, Sousse, Tunisia; ^4^ Technische Universität Chemnitz, Professorship Measurement and Sensor Technology, Chemnitz, Germany

**Keywords:** nanomaterials, biosensors, electrochemical detection, nanozymes, immunosensors

Electrochemical biosensors have shown tremendous potential in a wide range of biomedical, diagnostic, food-safety, and environmental applications because of their low cost, ease of use, and capability of miniaturization. With the recent advances in nanotechnology, considerable attention has been devoted to the use of nanoscale materials in biosensors (Wang 2005). Because of their extraordinary properties such as large surface area, high conductivity, high electrocatalytic activity, and fast electron transfer rate, nanomaterials have been extensively integrated into electrochemical biosensors. Nanomaterials offer excellent prospects for interfacing the biological recognition events with the electrochemical signal transduction leading to biosensing platforms with enhanced sensitivity and selectivity.

This Research Topic addresses the recent advances in various synthesis processes and properties of nanomaterials and their integration into electrochemical biosensing platforms. It covers advances in nanostructures and nanocomposites in terms of their synthesis, characterization, modifications, and applications in various enzymatic and affinity based-electrochemical biosensors. Novel nanomaterials improve the sensitivity, selectivity, and biocompatibility of the electrochemical devices and have potential applications on different areas such as food-safety, diagnosis, and environmental monitoring were covered.

Original research and review articles were gathered in this Research Topic covering the use of different nanomaterials such as snowflake Cu_2_S/Pd/CuO nanocomposite (Cao et al.), nanostructured gold (Wang et al.), reduced graphene oxide (Dey et al.) and Au modified PrFeO_3_ (Zhang et al.) in various biosensing applications such as food safety (Wu et al.), diagnosis of cancer (Cao et al.), Alzheimer (Wang et al.; Dey et al.) and periodontal diseases (Zhang et al.). Different biomarkers were targeted in these studies such as Carcinoembryonic antigen (CEA), Amyloid-β(1–42), β-Secretase Enzyme (BACE1) and H_2_S. Different characterization techniques of nanomaterial and detection approaches were presented. In particular:

Wu et al. have reviewed the various nanozymes and their use in the detection of food contaminants. They provided a comprehensive overview of the history, designs, principles, and applications of different nanozymatic analytical techniques for food safety applications. The authors detailed different modifications and engineering of nanozymes. Different detection methods were presented and discussed such as colorimetry, electrochemiluminescence, fluorescence, surface-enhanced Raman scattering, magnetic relaxing sensing, and electrochemical detection. Current challenges and future prospective of the applications of nanozymes in food safety were discussed.

Cao et al. have reported the detection of Carcinoembryonic antigen (CEA), one of the major tumor biomarkers used for diagnosis of colorectal cancer. Cu_2_S/Pd/CuO nanocomposite was synthesized *via* the *in situ* formation of Pd NPs on the Cu2S and was utilized to construct the electrochemical immunosensor. The nanocomposite was not only used as a carrier to increase the reaction area but also catalyzed the substrate to generate the current signal showing excellent catalytic characteristics. Moreover, the nanocomposite offered good biocompatibility and high electron transfer rate. The nanocomposite was used to fabricate a label-free immunosensor using H_2_O_2_ as the active substrate achieve very low detection limit for CEA. The biosensor was applied for the detection of CEA in human serum samples shown comparable results with those obtained by by commercial immunoassays.

Wang et al. have presented the detection of Amyloid-β(1–42) oligomer,a biomarker associated with physiologic changes in the brains of patients with Alzheimer’s disease, using sandwich electrochemical Immunosensor. The immunosensor was by immobilization of specific antibody on gold nanoparticles modified electrodes by self-assembly. Fluorescence microscopy and Western blot were used to confirm the presence of Aβ(1–42) monomers and oligomers on the biosensor surface. The electrochemical detection was realized by measuring the change in the resistance using electrochemical impedance spectroscopy. Different conformation of the Aβ(1–42) monomers and Aβ(1–42) oligomers were differentiated using the electrochemical biosensor. The biosensor showed high selectivity and sensitivity.

Dey et al. have employed reduced graphene oxide as electrode modifier to fabricate electrochemical biosensor for the detection of the Alzheimer’s Disease Biomarker, β-Secretase Enzyme (BACE1). The author showed the synthesis of reduced graphene oxide (rGO), its functionalization *via* carbodiimide chemistry, immobilization of BACE1 antibody on its surface using one-step process. Fluorine-doped tin oxide (FTO) electrodes were used as substrate for immobilized the functionalized antibody. Different characterization techniques such as microscopy, X-ray and voltammetry were used to characterize the synthesis of the nanomaterial and the stepwise fabrication of the biosensor. After optimization, the electrochemical immunosensor showed high selectivity, sensitivity, storage stability and repeatability. Moreover, the biosensor was successfully applied for the detection of BACE1 antigen in spiked serum and CSF.

Zhang et al. have reported the use of Au–PrFeO_3_ nanocrystalline powder for the detection of H_2_S, the gas found in the exhaled breath associated with the progress of periodontal disease. The high surface area and porosity of the nanomaterial prepared using an electrospinning method which led to high gold loading, provided sensitive detection of H_2_S. The doping of the material with Au adjusts the binding energy and decreases its resistance. This allows more oxygen ions to adsorb on its surface because of a spillover effect. The author showed the enhancement of the response of the Au–PrFeO_3_ by 10 times compared with the pure PrFeO_3_.

## References

[B1] Wang J. (2005). 'Nanomaterial-based electrochemical biosensors. Analyst 130, 421–426. 10.1039/b414248a 15846872

